# Development of a tomato xylem-mimicking microfluidic system to study *Ralstonia pseudosolanacearum* biofilm formation

**DOI:** 10.3389/fbioe.2024.1395959

**Published:** 2024-05-27

**Authors:** Lan Thanh Chu, Deeksha Laxman, Jenna Abdelhamed, Russell Kirk Pirlo, Fei Fan, Nicholas Wagner, Tuan Minh Tran, Loan Bui

**Affiliations:** ^1^ Department of Biology, University of Dayton, Dayton, OH, United States; ^2^ Department of Chemical and Materials Engineering, University of Dayton, Dayton, OH, United States; ^3^ Department of Chemistry, Michigan State University, East Lansing, MI, United States; ^4^ Department of Biology, University of South Alabama, Mobile, AL, United States

**Keywords:** xylem-mimicking, microfluidic, biofilm, *Ralstonia pseudosolanacearum*, carboxymethyl cellulose, polydopamine

## Abstract

The bacterial wilt pathogen *Ralstonia pseudosolanacearum (Rps)* colonizes plant xylem vessels and blocks the flow of xylem sap by its biofilm (comprising of bacterial cells and extracellular material), resulting in devastating wilt disease across many economically important host plants including tomatoes. The technical challenges of imaging the xylem environment, along with the use of artificial cell culture plates and media in existing *in vitro* systems, limit the understanding of *Rps* biofilm formation and its infection dynamics. In this study, we designed and built a microfluidic system that mimicked the physical and chemical conditions of the tomato xylem vessels, and allowed us to dissect *Rps* responses to different xylem-like conditions. The system, incorporating functional surface coatings of carboxymethyl cellulose-dopamine, provided a bioactive environment that significantly enhanced *Rps* attachment and biofilm formation in the presence of tomato xylem sap. Using computational approaches, we confirmed that *Rps* experienced linear increasing drag forces in xylem-mimicking channels at higher flow rates. Consistently, attachment and biofilm assays conducted in our microfluidic system revealed that both seeding time and flow rates were critical for bacterial adhesion to surface and biofilm formation inside the channels. These findings provided insights into the *Rps* attachment and biofilm formation processes, contributing to a better understanding of plant-pathogen interactions during wilt disease development.

## 1 Introduction

*Ralstonia solanacearum* species complex (*Rs*) is the major soil-borne bacterial pathogen causing the devastating vascular wilt disease in a wide range of host plants, among which are economically important crops such as tomato, potato, banana and pepper, etc. (Fegan et al., 2005; Weibel et al., 2016). *Rs* invades the root system through wounds or natural openings, then uses the xylem vessels as a highway to spread systemically within each plant and from plant to plant by graft inoculation. Inside xylem vessels as well as intracellular space, *Rs* forms complex biofilm, composed of bacterial cells and self-produced extracellular matrix (Mori et al., 2016; Tran et al., 2016; Khokhani et al., 2017). The accumulation of bacterial biofilm eventually blocks the sap flow, resulting in wilt symptoms (Ingel et al., 2021). It has been suggested that *Rs* biofilm formation is directly controlled by quorum sensing (Khokhani et al., 2017; Mori et al., 2018). However, biofilm-facilitated *Rs* virulence mechanisms inside the xylem remain unclear, such as how the bacterial cells adapt to the continuously flowing environment of the xylem, initiate attachment to the xylem wall, establish the founder microcolonies, and develop mature biofilm.

Xylem vessels are specialized structures that allow efficient transport of water and solutes from the root system to apical tissue. Tracheary elements are the main conductive tissue, accompanied by living xylem parenchyma cells. Once these tracheary elements undergo programmed cell death, their cellular content is cleared, leaving behind hollow tubes that make up xylem vessels (De La Fuente et al., 2022). Physical structures and chemical components of the xylem vessels vary depending on the cultivars, plant age and tissue types (Kim et al., 2014; Jacobsen et al., 2018). In tomato, xylem cross-section areas in the roots of different cultivars range from 200 to 1,600 μm^2^ (Caldwell et al., 2017); and the diameters of embolized vessels in the stems range from 10 to 60 µm (Khokhani et al., 2017; Ingel et al., 2021). Chemically, the xylem cell wall of tomato consists mainly of cellulose and hemicellulose (Seymour et al., 1990; Zhang et al., 2021; Frey et al., 2022). Other polysaccharides such as lignin and pectin were found to increase in maturing vessels (Lowe-Power et al., 2018) and in wilt resistant cultivars (Ishihara et al., 2012; Kashyap et al., 2022). Most *in vitro* studies of *Rs* biofilm, however, were conducted on glass or plastic surfaces such as PVC plates, which may not represent the relevant conditions *Rs* experiences in the xylem. In addition, since xylem is located deep inside plant tissue, *in vivo* studies of xylem-inhabiting pathogens rely on dissecting infected plants, which do not allow for real-time and long-term monitoring of bacterial biofilms throughout the course of infection.

Microfluidic technology has emerged as a versatile tool for creating lab-on-chip systems, shedding light on the intricate interactions between plants and bacteria at both cellular and subcellular levels within flowing micronetworks. Initially, simple microfluidic devices were developed as pioneering platforms for exploring how drag forces influence the adhesiveness of *Xylella fastidiosa*, the bacterium causing Pierce’s disease in grapevines (De La Fuente et al., 2007). Building on this groundwork, further studies employed these channels to mathematically analyze the distinctive striking patterns generated by the same bacterium (Cogan et al., 2013). Enhancements in bioactivity within microfluidic systems can be achieved by integrating plant parts or simulating specific plant structures and plant tissue environments. For instance, a device engineered by Massalha et al. (2017) accommodated *Arabidopsis thaliana* roots, facilitating research on their interactions with *Bacillus subtilis*. Additionally, more sophisticated designs introduced interactive channels and pectin-rich simulated xylem media, enabling investigations into the dynamics between *Pseudomonas* *protegens* and the *Verticillium* sp., a fungal pathogen causing vascular wilt diseases (Harting et al., 2021). The system’s complexity was further achieved by adding cell wall components and bacterial adhesins, like the *Xylella fastidiosa* adhesin XadA, as a coating, enabling studies of both plant and bacterial factors in the biofilm formation process of xylem pathogens (Monteiro et al., 2021).

**FIGURE 1:**
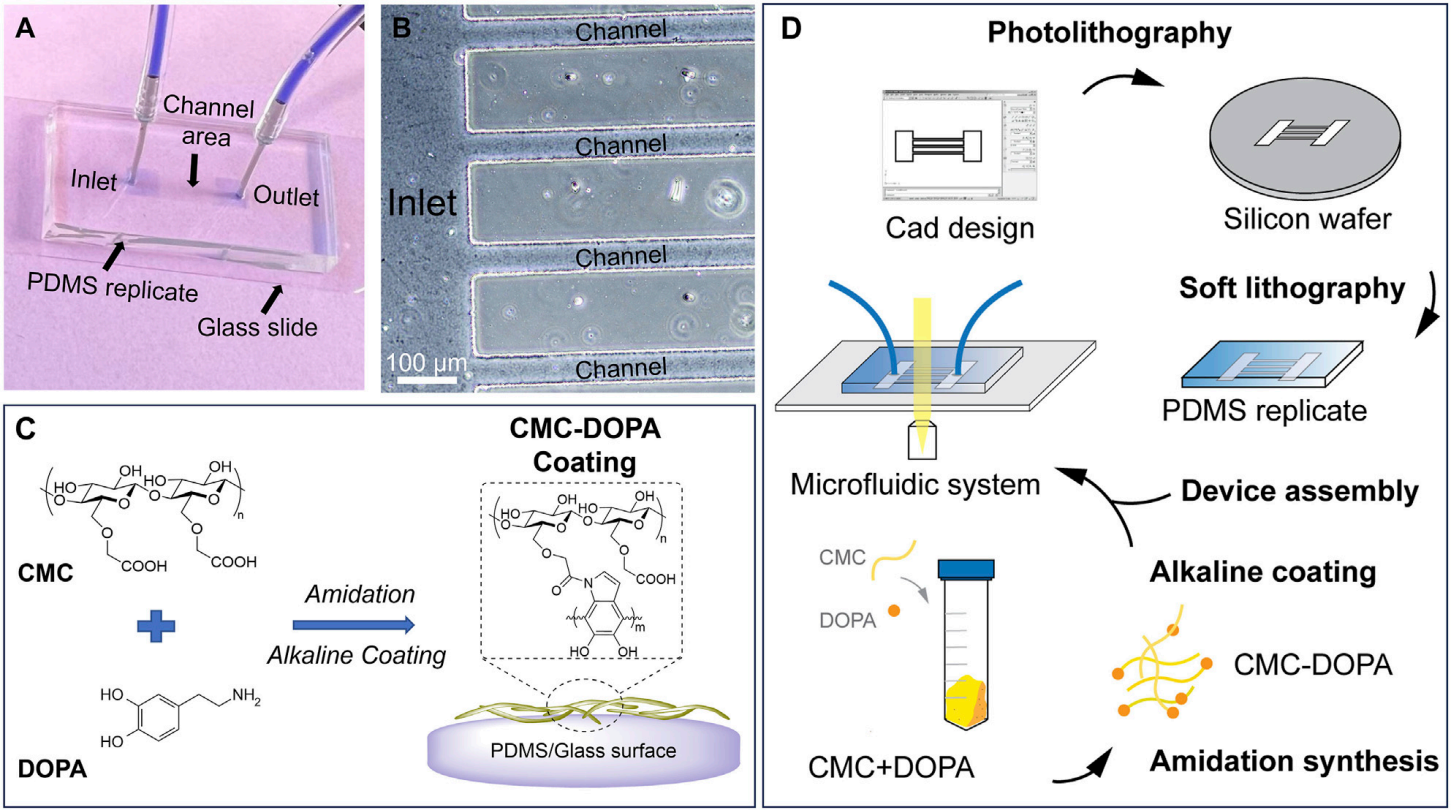
A microfluidic system mimicking tomato xylem for studying biofilm formation. **(A)** A PDMS microfluidic system assembled on a glass slide with inlet and outlet reservoirs (two blue squares) connected by channels in the middle. **(B)** A zoomed-in image showing part of the inlet reservoir and channels. **(C)** Cell-wall mimicking coating for the channels: carboxymethyl cellulose (CMC) reacts with dopamine (DOPA) via amidation. Under an alkaline condition, CMC-DOPA forms a coating on PDMS and glass surfaces. **(D)** Schematics of microfluidic system preparation.

In this study, using microfluidic technology and biomaterial mimicry, we establish a novel bioactive platform that allows us to dissect different stages of *Ralstonia pseudosolanacearum* (representing Phylotype I and III of *Rs*) biofilm formation under flow conditions. We focus on developing a microfluidic system with channel dimensions and flow characteristics mimicking tomato plant xylem environment. Note that changes in the flow characteristics such as the shear rate and the wall shear stress can significantly affect the bacteria motility and their attachment mechanisms (Yuan et al., 2023). Such changes are directly attributed by varied xylem sap viscosities due to temperatures or cultivars, and xylem sap flow rates due to different water transpiration and photosynthesis between day and night (Michelle Holbrook and Zwieniecki, 2011; Lowe-Power et al., 2018). We then enhanced the bioactivity of the channels with carboxymethyl cellulose (CMC) via polydopamine surface chemistry. CMC, a synthetic derivative of cellulose, mimics cellulose, the main component of xylem cell walls (Figures 1C, D). Dopamine, or 3,4-dihydroxyphenethylamine (DOPA), is an inspiring biomaterial found from mussels’ adhesive proteins. Via amidation with CMC and self-polymerization, the resulting CMC-DOPA forms a thin and surface-adherent coating with many substrates including glass and PDMS (Barclay et al., 2017). The final microfluidic system integrates with microscopes that allow visualization and quantification of multiple bacterial behaviors within the dynamic flow within the channels (Figure 1D).

## 2 Methodology

### 2.1 Design and fabrication of the microfluidic system

To design the microfluidic system, we used AutoCAD^®^ as the computer-aid tool. The system consists of 20 parallel channels with a constant width of 50 µm and length of 7 mm. There are square inlet and outlet reservoirs (4 × 4 mm) located at two ends of the channels. The design was sent for transparent mask fabrication (Fine Line Imaging, 50,000 dpi). By conducting standard photolithography technology (Bui et al., 2017; Bui et al., 2018; Hobbs et al., 2021), we generated a silicon mold (University Wafer, 3,774) with SU8-2025 negative photoresist features (MicroChem). The photoresist was spin-coated on a silicon wafer at 1,800 rpm for 30 s, then soft-baked at 65°C for 3 min and 95°C for 10 min. The photoresist in hard-contact with the mask was exposed for 10 s (KLOE UV-KUB 2, Power density 52.3 mW/cm^2^), post-baked at 65°C for 2 min, and 95°C for 10 min. The channel features appeared after being developed for 5 min. The desired thickness of the resulting photoresist was 50 μm, defining the height of the channels and the reservoirs. These features were replicated into PDMS (Dow Corning, SYLGARD™ 182 Silicone Elastomer Kit) via soft lithography technique (Figures 1A, B, D) (Bui et al., 2017; Bui et al., 2018; Hobbs et al., 2021).

To assemble a microfluidic system for experiments, the PDMS replicates, glass slides, tubings and connectors were decontaminated with ethanol 70% and washed three times with sterile deionized (DI) water inside a biosafety hood. Then the PDMS replicate was assembled to a glass slide to form closed channels. For achieving irreversible attachment and preventing fluid leakage, prior to assembly, both PDMS and the glass slides were ozone-treated for 5 min (Novascan, PSD Series System) (Bui et al., 2016; Bui et al., 2017). Tubings and connectors were also assembled to the inlet and outlet reservoirs. For flow study, the inlet was connected to a syringe pump (Fisherbrand™, 780100) to control the fluid movement through the channels.

### 2.2 Characterization of the flow within the channel

Flow rate through the system inlet was set in the syringe pump (Fisherbrand™, 780100) at 40 μL/h. The flow was monitored by introducing 2 µm fluorescence polymer microspheres (R0200, Thermo Scientific) as tracer particles in low concentration (0.06% v/v), dispersed in DI water (Mina et al., 2016; Pollet et al., 2019). Videos of the tracers in flow at the channel’s entrance, middle and end were recorded in three channels (1, 10, 20). All videos were taken at ×20 objective for 10 s using a fluorescent microscope (Nikon Eclipse Ts2), and saved as TIFF files for analysis. The trajectories and velocities of the microspheres within the channels were analyzed using the Trackmate plugin (Mina et al., 2016; Pollet et al., 2019) in Fiji. Briefly the files were first converted into 8-bit and scale was set in micron. Using Trackmate, a thresholding detector was applied with the intensity adjusted from 30 to 40 to define individual microspheres. Once all microspheres in all frames were detectable, we then utilized the simple LAP tracker, the tool specific for non-branching tracks, to quantify the lateral (XY) trajectories and the mean velocity of the microspheres (Tsvirkun et al., 2017; Dubay et al., 2023). The plugin compiled all successive positions, constructed the track list, and exported data to an excel file for further analysis.

The flow profile within the microfluidic system was also computationally analyzed using COMSOL Multiphysics^®^ software. A single channel with a square cross-section of 50 μm × 50 μm and a length of 7 mm was generated to study variations in velocity, shear stress, and drag force. The simulation was run using the Laminar Flow module and Stationary study. Water was used as the fluid material; and a constant flow rate of 2 μL/h (i.e. 5.56 × 10^−13^ m^3^/s for water) was set at the inlet boundary condition (equivalent to the flow rate of 40 μL/h imposed by the syringe pump). The fluid dynamics inside the channel is governed by the following default Navier-Stokes equations in COMSOL for incompressible flow, where ρ is the density, u is the velocity vector, p is the pressure, μ is the dynamic viscosity, T is the temperature, and F is the external force applied on the fluid. Then, a fine meshing system for the channel was used to perform the simulation. Heatmap diagrams of the fluid velocity throughout the channel were generated by the software.

$$\rho \left(u\cdot \nabla \right)u=\nabla \cdot \left[-pI+\mu \left(\nabla u+{\left(\nabla u\right)}^{T}\right)\right]+F$$

ρ∇⋅u=0

For a Newtonian fluid like water, shear stress (σ_s_), which represents the tangential force per unit area exerted by the flowing fluid on a surface, is directly proportional to the shear rate (γ). This shear stress profile within the channel can be calculated and graphed in COMSOL via the following default equation.

$$\sigma_{s}=\mu \gamma$$

The shear stress also attributes to the drag force applied on the bacteria that reside on the channel or move within the flow. Once the drag force overcomes the adhesive force, it removes adherent bacteria from the surface. To analyze the drag forces applied on a bacterium, we added a bacterial cell model with a diameter of 0.7 μm and an end-to-end length of 2.9 μm at the mid-wall of the channel (Figure 4C). The drag force (F_drag_) was estimated using the equation below, where A indicated the area of the bacteria in contact with the fluid flowing through the channel; and σ_s_ denoted the shear stress acting on the bacterium (Persat et al., 2015; Yuan et al., 2023).

$$F_{\text{drag}}=A\sigma_{s}$$

### 2.3 Preparation of CMC-DOPA coating for channel surface modification

Synthesis of CMC-DOPA was performed via amidation reaction (Wang et al., 2021; Bian et al., 2023). Sodium carboxymethyl cellulose with 0.7 carboxymethyl groups per anhydroglucose unit (CMC, Sigma-Aldrich, 419273) was dissolved in 0.1 M MES buffer (pH 5.5) at concentration of 10 mg/mL. Pure ethanol (Sigma-Aldrich, 4450-500ML) was added at a ratio of ethanol:MES buffer 1:3 (v/v). Then, 1 M equivalent of 4-(4,6-Dimethoxy-1,3,5-triazin-2-yl)-4-methylmorpholinium Chloride (DMTMM, TCI America, TCD2919-5G) was introduced to activate the carboxyl group for 15 min, followed by introduction of 0.7 M equivalent of Dopamine hydrochloride (DOPA, Sigma-Aldrich H8502-25G). The mixture was protected from light and stirred under room temperature for 1 day, then purified by dialysis against 1% NaCl solution and DI water for 2 and 3 days, respectively. The CMC-DOPA product was lyophilized using a freeze dry system (Labconco FreeZone 6) and stored at −20°C until use.

Chemical structure of CMC-DOPA was evaluated by ^1^H-NMR (Wang et al., 2021). Approximately 3 mg of CMC-DOPA was dissolved in Deuterium oxide (D_2_O, Sigma-Aldrich, 1.13366.0025) for hydrogen-to-deuterium exchange, followed by freeze drying then dissolving it again in D_2_O. ^1^H-NMR was conducted with a Bruker Ascend™ 400 MHz NMR Spectrometer and data was analyzed by MNova software. The degree of substitution (DS) of DOPA, which represents the percentage of anhydroglucose in CMC containing DOPA, was quantified by UV-Vis spectrometry at 280 nm. A standard curve of different DOPA concentrations was first generated; and absorbance of the solutions was read using UV-Star^Ⓡ^ 96 well plates (Greiner Bio-One 655801) and a plate reader (BioTek, Synergy LX). Then CMC-DOPA was diluted in DI water, followed by measuring the absorbance. The DS value of DOPA in CMC-DOPA was calculated based on the absorbance measurement and standard curve.

The CMC amounts on the resulting CMC-DOPA were quantified via methyl blue assay (Yan and Chai, 2021). To create a standard curve, different solutions of CMC (0–100 mg/L) were prepared in DI water and mixed with 20 mg/L methyl blue solution at 1:1 v/v ratio. Methyl blue absorbance of all mixtures at 597 nm were measured on 96 well plates (100 µL per well); then the inverse of the absorbance values was used to generate the standard curve to show the linear effect of CMC on interfering the absorbance (data not shown). Absorbance spectra of methyl blue solution alone (MB) and when mixed with CMC (50 mg/L), CMC-DOPA (50 mg/L) and DOPA (1 mg/L, equivalent to the DS value) were measured.

The coating on the PDMS or glass surface was conducted under alkaline conditions. CMC-DOPA was dissolved in DI water to the desired concentrations (e.g., 5, 10, 20 mg/mL). The solution was alkalinized by adding 20 μL of 1 N NaOH per 1 mL of the CMC-DOPA solution. Coating solution was introduced onto plain PDMS and glass surfaces (static condition) or run through the channel using a syringe pump (Fisherbrand™, 780100) with low flow rate (10 μL/h) for 60 min with 5 min interval. The coated surfaces were incubated at 37°C overnight, followed by washing with DI water three times (static condition) or with PBS (flow condition) running through the channel for 2 h at 10 μL/h flow rate.

### 2.4 Characterization of CMC-DOPA and coated surfaces

The coated surfaces were quantified via Calcofluor White stain (CFW) assay (Krause and Olsen, 2020). To create a standard curve, different CMC concentrations (0–200 mg/L) were prepared in DI water. CFW (Sigma-Aldrich, 18909) was diluted at 1:10 v/v ratio in DI water to make a staining solution. Then 100 µL CMC solution was mixed with 40 µL CFW staining solution on top of a well with either a glass or PDMS bottom. The mixture was incubated at room temperature for 15 min then imaged at ×10 objective using DAPI channel (Nikon Eclipse Ts2) with exposure time at 200 ms and 1X gain (*n* = 4 per concentration). All images were quantified for mean fluorescent intensity values using Fiji. The average of mean intensity values at each concentration was calculated and normalized to the values of 0 mg/L concentration in order to generate a standard curve (Supplementary Figure S1). The surfaces coated with CMC-DOPA (0, 5, 10, 20 mg/mL) were prepared and washed as above-mentioned. 100 μL DI water and 40 µL CFW staining solution were added onto the coated surfaces (*n* = 6–13 per concentration), followed by incubation and imaging. The resulting fluorescent intensity on each coated surface was quantified. Based on the standard curve, the amount of CMC on the coated surface was calculated.

The wettability of the coated surfaces was quantified via contact angle measurement using a Kruss DSA100 Drop Shape Analyzer. Glass and PDMS surfaces were coated with CMC-DOPA concentrations of 0, 5, 10, and 20 mg/mL, as described above. After rinsing with DI water, the surfaces were allowed to dry for 10 min inside a biosafety hood. A 20 µL volume of DI water was deposited onto each surface; the droplets were then imaged and analyzed using the DSA software. The sessile drop method was applied to measure left and right contact angles of the samples as well as the mean of the two angle values. Ten mean measurements for a single droplet were then recorded, and each coating concentration was assessed in four replicates. The results were plotted and statistical differences between the coated surfaces and the control (0 mg/mL) were determined using Excel and GraphPad.

### 2.5 Plant growth condition and xylem sap collection

Tomato cv. Bonny Best (wilt susceptible) and cv. Hawaii 7996 (wilt resistant) plants were grown at 28°C with a 12 h light/12 h dark photoperiod. Xylem sap was collected from five-week-old plants by decapitating the plants above the cotyledons. The cut stems were wiped with Kimwipes to remove phloem sap and cell debris. The xylem sap was collected for a period of 3 h, kept on ice, then filter-sterilized using 0.22-µm filters and frozen at −80°C until use (Khokhani et al., 2017).

### 2.6 Growth media, bacterial culture conditions, and biofilm assays

#### 2.6.1 Bacterial culture and maintenance

*R. pseudosolanacearum* strain GMI1000 (hereby, *Rps*) was maintained regularly on Cassamino acids-Peptone-Glucose (CPG) broth or solid at 28°C (Kelman, 1954). *Rps* GMI1000 GFP strain (Tran et al., 2016) was maintained on CPG + Tetracycline (15 μg/mL).

#### 2.6.2 Biofilm assays on PVC plates and on coated substrates (glass or PDMS)

*Rps* was grown in liquid CPG overnight and adjusted to OD_600nm_ = 0.1 in either CPG, Bonny Best xylem sap, or Hawaii 7996 xylem sap. Biofilm was grown for 24 h at 28°C, then the bacterial culture was removed from the PVC wells or from the coated surfaces. The biofilm was stained with 25 µL of 1% Crystal Violet (Thermo Fisher, C581-100) at room temperature for 25 min, then washed three times with sterile DI water to remove free cells and unbound Crystal Violet. To quantify biofilm formed on PVC plates, Crystal Violet was dissolved in 200 µL of 95% ethanol and transferred to a new polystyrene plate and the absorbance was measured at 590 nm using a Synergy H1 microtiter plate reader (Biotek Instruments). To visualize biofilm formation on the coated surfaces, *Rps* biofilm was imaged after washing using a compound light microscope equipped with a microscope camera (Amscope). Biofilm formation on glass and PDMS surfaces was quantified by analyzing the percentage of surface coverage using Fiji’s “Analyze particles” plug-in. The biofilm experiments on PDMS and glass surfaces were repeated once and twice, respectively, with biofilm in each concentration of CMC-DOPA imaged at at least 10 different positions for each coating concentration.

#### 2.6.3 *Rps* attachment and biofilm formation in microfluidic devices

After coating the channel with CMC-DOPA (see coating procedure above), a suspension of 10^9^ CFU/mL was run through each device until the suspension fully filled the channels, then the devices were incubated for 6 h at room temperature. CPG medium or tomato xylem sap were injected into the microfluidic devices by a syringe pump at predetermined flow rates depending on experiments. Unless specified, the flow rate was set at 40 μL/h (similar to flow rate of xylem sap measured in Bonny Best tomato plants). The microfluidic experiments were carried out for 3 days at room temperature, with media replenished every 24 h. On the third day, bacterial biofilm in the microfluidic devices was stained with 1% Crystal Violet, then washed three times with sterilized DI water to remove planktonic cells and excess Crystal Violet before imaging with a compound light microscope equipped with an Amscope camera. Surface coverage was quantified using Fiji. For bacterial attachment, GFP-expressing *Rps* GMI1000 cells were introduced into microfluidic devices at different flow rates or seeding time, then planktonic cells were flushed from the channels with water before imaging using a Zeiss LSM 980 Airyscan system equipped with a Plan-Apochromat ×63 oil objective (NA = 1.40). These experiments were performed once. Data shown are representative of twenty channels per device.

## 3 Results

### 3.1 CMC-DOPA expressed unique chemical properties of both CMC and DOPA

**FIGURE 2:**
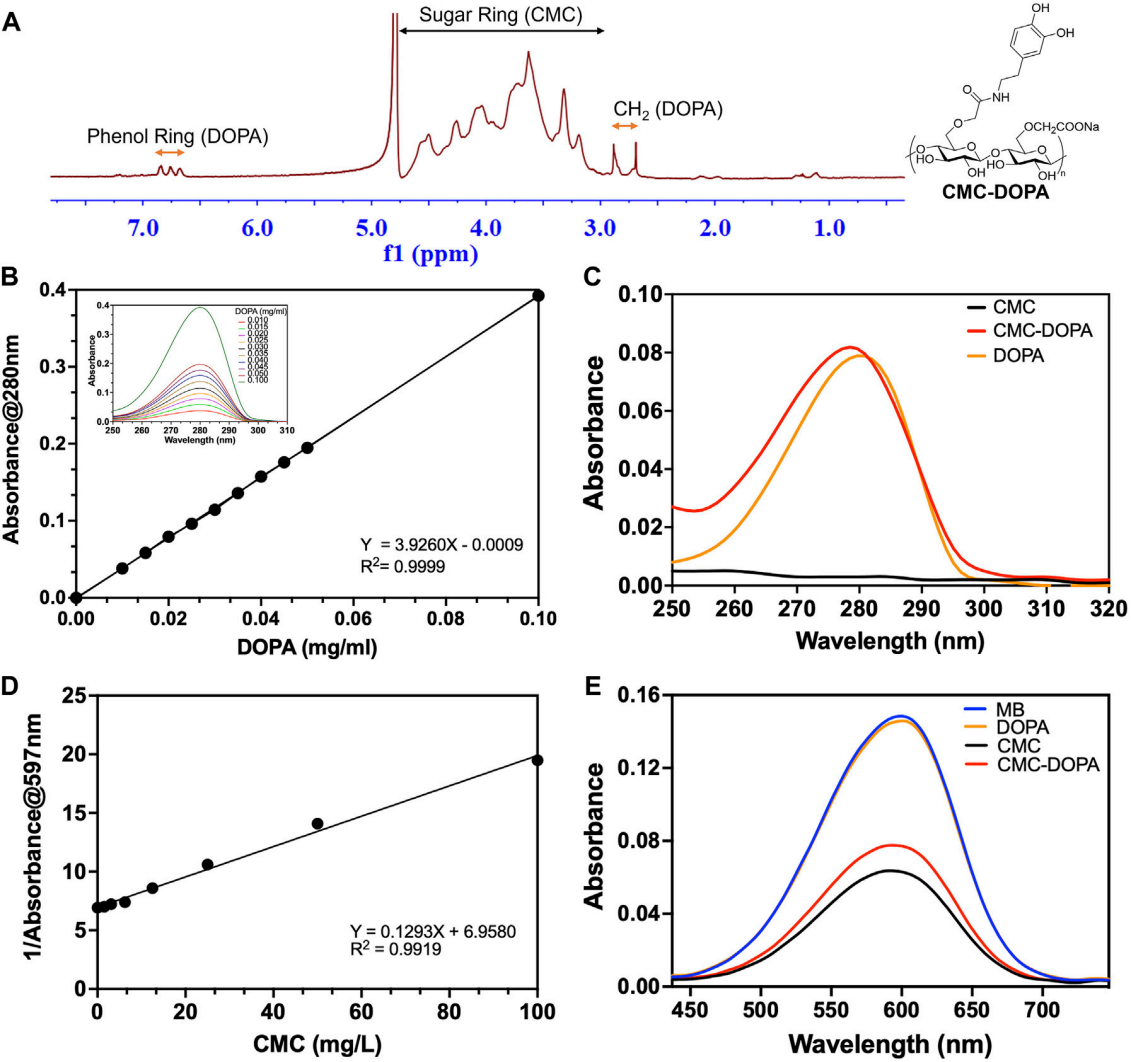
Synthesized CMC-DOPA with distinct ^1^H NMR and absorbance spectra. **(A)** ^1^H NMR spectrum showing successful synthesis of CMC-DOPA with distinct phenol ring and CH_2_ peaks (DOPA) and sugar ring signals (CMC). **(B)** Standard curve of different DOPA concentrations generated from absorbance measurements at 280 nm and absorbance spectra of DOPA with detected peaks at 280 nm (inset). **(C)** Absorbance spectra of CMC (1 mg/mL), CMC-DOPA (1 mg/mL) and DOPA (0.02 mg/mL). Both CMC-DOPA and DOPA expressed absorbance peaks at 280 nm. **(D)** Standard curve of different CMC concentrations generated from the inverse of the methyl blue absorbance measurements at 597 nm. **(E)** Absorbance spectra of methyl blue solution alone (MB) and when mixed with CMC (50 mg/L), CMC-DOPA (50 mg/L) and DOPA (1 mg/L). Both CMC and CMC-DOPA showed comparable reduced absorbance values.

The chemical structure of the synthesized CMC-DOPA was characterized by ^1^H NMR (Figure 2A). The ^1^H NMR spectrum revealed distinct peaks equivalent to the sugar ring of CMC (3.00–4.75 ppm), the CH_2_ group (2.80 ppm) and the catechol group (dihydric phenol, 6.75 ppm) of DOPA (Wang et al., 2021). The presence of DOPA in the synthesized product was further demonstrated via the similar UV absorbance spectra and characteristic peaks of the catechol group at 280 nm observed in DOPA and CMC-DOPA (Figure 2C) (Chen et al., 2019). Also, using the absorbance data, we were able to generate a standard curve of DOPA concentrations (Figure 2B) and calculated the DS value of the synthesized CMC-DOPA, which was 2.53 (±0.16) % (*n* = 4 batches) and consistent batch to batch. Finally, as CMC showed its capability to linearly interfere with methyl blue (MB) absorbance via ionic association [Figure 2D, (Yan and Chai, 2021)], we then examined if CMC-DOPA retained CMC property by comparing the spectra of methyl blue solution alone and its mixture with either DOPA (1 mg/L), CMC (50 mg/L), or CMC-DOPA (50 mg/L). As a result, the DOPA did not cause notable changes in the MB absorbance spectrum while both CMC and CMC-DOPA reduced the absorbance significantly (Figure 2E). Note that the CMC-DOPA had less CMC compared to the CMC at the same concentration, which may affect the slight difference between the two spectra.

### 3.2 CMC-DOPA coating functionalized surface substrates for *Rps* biofilm formation

**FIGURE 3:**
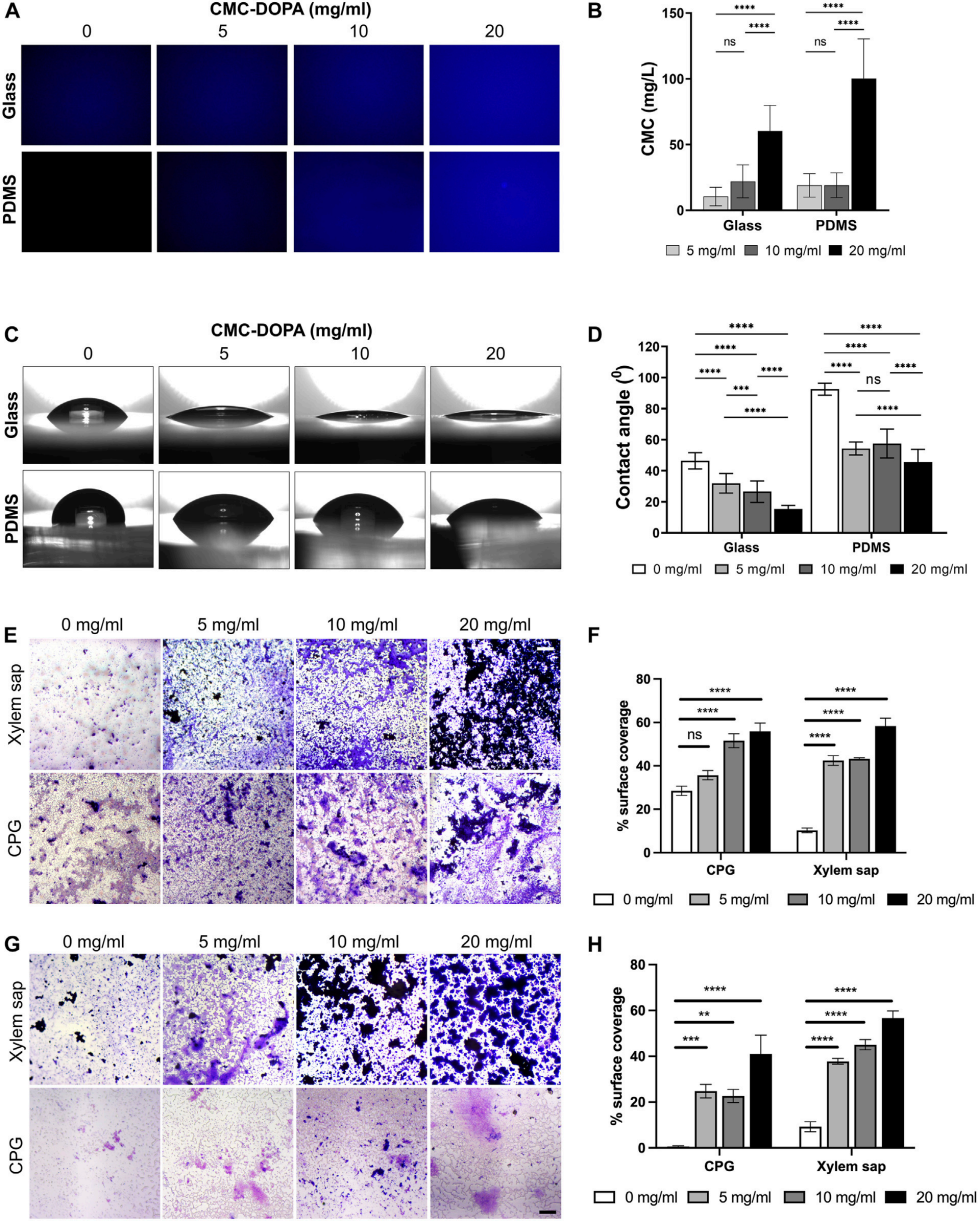
Functionalization of channel surfaces via CMC-DOPA coating. **(A)** Glass and PDMS surfaces coated with CMC-DOPA at different concentrations evaluated by CFW fluorescent imaging, and **(B)** quantified by mean fluorescent intensity. **(C)** Wettability assessed via water drops on glass and PDMS substrates coated with different CMC-DOPA concentrations. **(D)** Increased wettability of the coated surfaces quantified by water droplet’s contact angles. **(E)** *Rps* biofilm stained with Crystal Violet on CMC-DOPA-coated glass surfaces (scale bar, 100 µm). **(F)** CMC-DOPA coated glass surfaces (0, 5, 10, 20 mg/mL) increased *Rps* biofilm formation in CPG medium and Bonny Best xylem sap. **(G)** *Rps* biofilm stained with Crystal Violet on CMC-DOPA-coated PDMS surfaces (scale bar, 100 µm). **(H)** CMC-DOPA coated PDMS surfaces (0, 5, 10, 20 mg/mL) increased *Rps* biofilm formation in both CPG medium and Bonny Best xylem sap (scale bar, 100 µm).

To assess the functionality of the CMC-DOPA coated surfaces, we first determined the coating efficiency on glass and PDMS substrates with different CMC-DOPA concentrations via Calcofluor White (CFW) staining and fluorescent image quantification. Note that the mean fluorescence intensity of CFW expressed a direct linear correlation with the increasing concentration of CMC (Supplementary Figure S1) due to the specific binding of CFW to cellulose (Wang et al., 2017; Deng et al., 2020; Krause and Olsen, 2020), which allowed us to directly deduce the CMC content on CMC-DOPA coated surfaces. As the result, we showed increases of detected CMC, 10.45 (±7.05), 22.06 (±12.57), 60.41 (±19.33) mg/L on glass and 18.95 (±8.91), 19.08 (±9.41), 100.13 (±30.25) mg/L on PDMS, with respect to increased CMC-DOPA coating concentrations of 5, 10, 20 mg/mL (Figures 3A, B). The CMC detected at 20 mg/mL was significantly higher than the other concentrations whereas between 5 and 10 mg/mL, there was no significant difference (Figure 3B). Finally, smaller standard deviations on glass surfaces might indicate a more uniform coating on the glass than on PDMS substrate.

The water contact angle test was utilized to assess the wettability of glass and PDMS substrates with and without CMC-DOPA coatings. As the CMC-DOPA concentration increased from 0 to 20 mg/mL, significant changes were observed in the average contact angles (Figures 3C, D). For the glass substrate, the water contact angle decreased from 46.38 (±5.18)° to 15.41 (±2.34)° as CMC-DOPA concentration increased from 0 to 20 mg/mL. Similarly, for the PDMS substrate, the water contact angle decreased from 92.53 (±3.85)° to 45.56 (±8.20)°. These results suggest that CMC-DOPA provided a robust adherent coating for both glass and PDMS substrates and the resulting coated surfaces became more hydrophilic. Note that the contact angle on the PDMS substrate was higher than that on glass due to the inherently higher hydrophobicity of PDMS as compared to glass (Yeo et al., 2006; Morent et al., 2007). In addition, the average of contact angle on PDMS surfaces coated with 5 mg/mL CMC-DOPA, 54.32 (±4.10)°, was slightly lower compared to those coated with 10 mg/mL, 57.58 (±9.31)°, a difference not observed on glass surfaces. There was also no significant difference between 5 and 10 mg/mL CMC-DOPA coatings on PDMS substrates (Figure 3D). Due to the hydrophobicity of PDMS, this phenomenon could be attributed to the coating baseline effect on PDMS at low CMC-DOPA concentrations (10 mg/mL or lower).

We observed that *Rps* formed biofilm poorly on glass and plastic surfaces such as PVC when it grew in xylem sap, even though this is the native condition *Rps* thrives in *in planta* (Supplementary Figure S2). To evaluate the effect of functionalized coatings on improving *Rps* biofilm formation, we coated the glass surface with 0, 5, 10, 20 mg/mL CMC-DOPA and imaged biofilm formation of *Rps* GMI1000 on these coated surfaces. We observed that while *Rps* formed thick biofilm that covered a large portion of the well surfaces in CPG, without CMC-DOPA coating, *Rps* only formed small microcolonies that covered approximately 10% of the surface in the presence of Bonny Best xylem sap (Figures 3E, F). Coating of glass surface with CMC-DOPA significantly enhanced *Rps* biofilm formation (Figure 3F), indicated by the increase in surface coverage of *Rps* biofilm stained with Crystal Violet (Figure 3E). At 20 mg/mL, CMC-DOPA coating increased the *Rps* biofilm surface coverage by approximately six times compared to control (no CMC-DOPA coating). Similar to coated-glass surfaces, we also found that on CMC-DOPA-coated PDMS, increasing the concentration of CMC-DOPA coating enhanced *Rps* biofilm surface coverage (Figures 3G, H). Interestingly, on the PDMS surface, the Crystal Violet-stained *Rps* biofilm appeared to be thicker, but more spatially constricted in the presence of xylem sap. In contrast, *Rps* formed a thin layer of biofilm covering the bottom of the PDMS wells when the bacterium was grown in CPG broth. These results showed that we have successfully developed a functionalized surface coating with CMC-DOPA, facilitating *Rps* attachment to surfaces and biofilm formation in the bacterium’s native condition: the tomato xylem sap environment.

### 3.3 *Rps* underwent laminar flow and physical force within the xylem-mimicking channels

**FIGURE 4:**
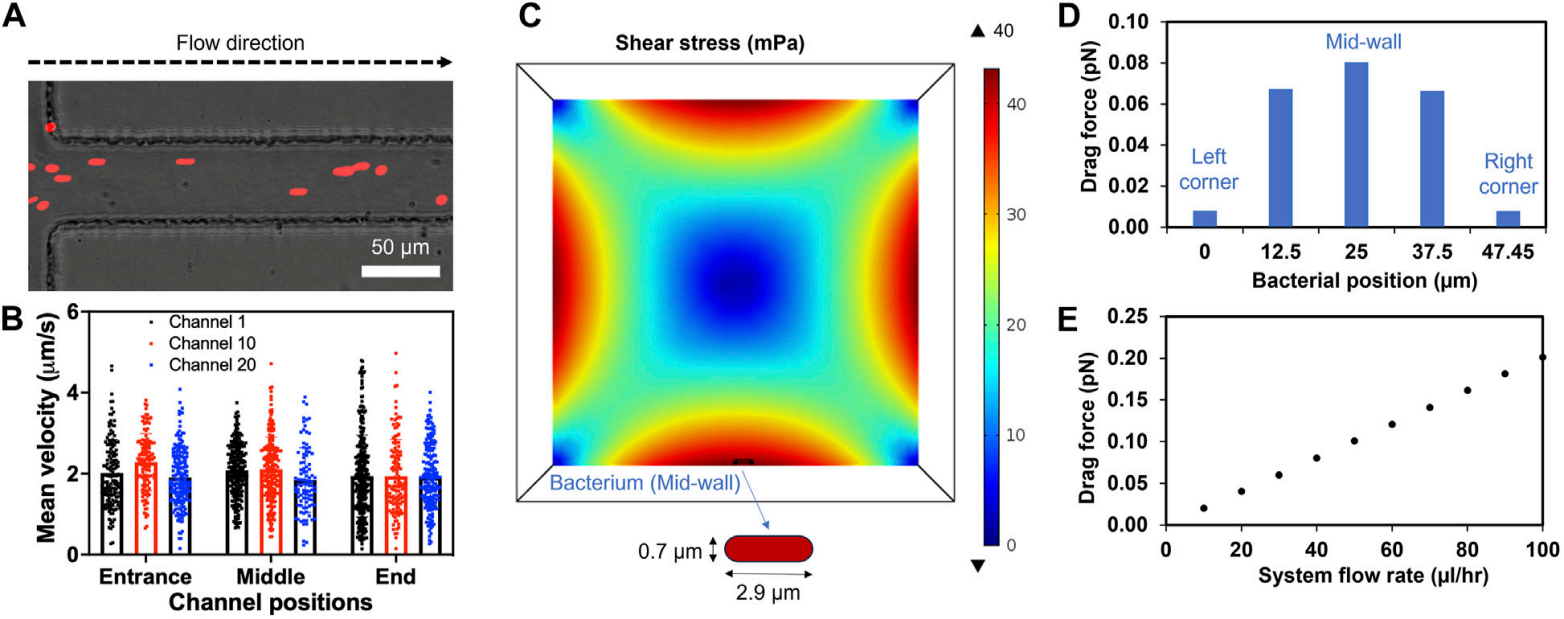
Laminar flow in the channels and drag forces applied on the bacterium model. **(A)** A representative image showing microspheres (Red, 2 µm in diameter) entering and flowing inside the channel. **(B)** Mean velocity profiles of the microspheres in three different channels calculated using Fiji (Trackmate plugin). **(C)** Cross sectional shear stress computationally calculated at the center line of a bacterial cell placed at the middle of the bottom wall (mid-wall) of the channel. **(D)** Changes of drag force applied on the bacterium moving from left to right corner. **(E)** The drag force applied on the bacterium at the mid-wall showing linear relation with increased system flow rates. System fluid flow rate preset at 40 μL/h (equivalent to each channel flow rate of 2 μL/h).

The flow profile and its effect on a bacterium in a microchannel were examined both computationally and experimentally. The system flow rate was set as 40 μL/h based on the average xylem sap flow rate of 38.3 (±4.4) μL/hr measured directly from Bonny Best plants (Supplementary Figure S3). DI water was used as the fluid due to its similar viscosity to xylem saps and culture media (Supplementary Figure S4). The simulation confirmed uniform velocity profile throughout the length of the channel (Supplementary Figure S5A). The flow velocity followed the Hagen-Poiseuille parabolic profile with highest values in the center (4.86 μm/s) and smaller towards the channel wall. Moreover, 2 μm microspheres were introduced to the inlet of the xylem-mimicking channels to visualize the flow experimentally (Figure 4A). The microsphere movement through three channels (channel 1, 10, 20) at three different locations (entrance, middle and end) was monitored and quantified. The result showed that the mean velocities of microspheres were comparable among three channels: 2.00 (±0.87), 2.11 (±0.81), and 1.90 (±0.75) μm/s in channel 1, 10, 20, respectively. Also, the velocity ranges were uniform across three channels regardless of channel’s locations and were consistent with the simulation results (Figure 4B; Supplementary Figure S5A). For further understanding the effect of fluid flow on *Rps*, a bacterial cell model with diameter of 0.7 μm and an end-to-end length of 2.9 μm was introduced to the bottom wall of the channel (Figure 4C). We noticed that the varying shear stress values due to the fluid flow (Supplementary Figure S5B) affected the drag forces applied on the bacterium model. When the bacterium model was placed at the mid-wall (Figure 4C), where the simulated shear stress was found maximum, the drag force was observed to be highest (0.08 pN). As we gradually moved the bacterium model toward the corners of the channel, drag force also decreased (Figure 4D). In addition, when we increased the system flow rate from 0 to 100 μL/h, the drag force also increased linearly from 0 to 0.2 pN (Figure 4E) indicating a linear relationship between the system flow rate and the drag force.

### 3.4 Flow rates within the xylem-mimicking channels influenced *Rps* surface adhesion and biofilm formation

**FIGURE 5:**
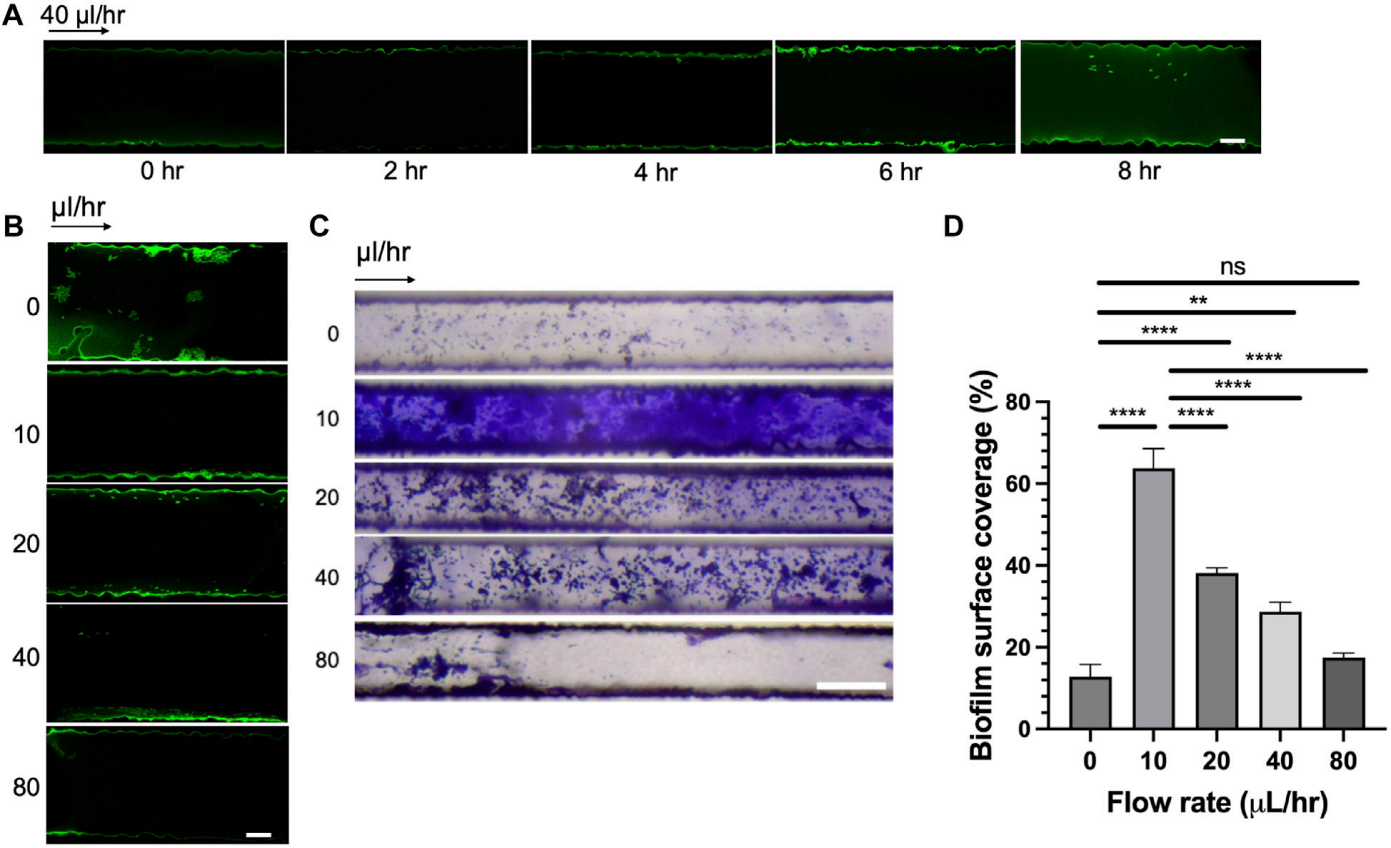
Effect of seeding time and flow rates on bacterial adhesion and biofilm formation. **(A)** *Rps* GMI1000 GFP adhesion in microfluidic channels over time (scale bar, 10 µm). **(B)** *Rps* GMI1000 GFP adhesion to microfluidic channels at different flow rates. *Rps* cells were seeded for 6 h in the devices, then flow was introduced at different rates (scale bar, 10 µm). **(C)** Biofilm formation of *Rps* GMI1000 in microfluidic devices after 3 days at different flow rates from 0 to 80 μL/h as visualized by Crystal Violet staining (scale bar, 50 µm). Arrows indicated the direction of xylem sap flown into the microfluidic devices. **(D)** Quantification of biofilm surface coverage from **(C)**. Surface coverage was calculated by area covered by Crystal Violet-stained biofilm divided by total area of the channel image.

To dissect the effect of seeding time and flow rates on bacterial attachment, we incubated CMC-DOPA coated channels with GFP-expressing *Rps* cells and monitored the attachment of bacterial cells in the microfluidic devices over a period of 8 h. We found that within the first 4 h, there was minimal attachment of *Rps* cells onto the surface of the channels (Figure 5A). At 6 h, it was evident that *Rps* cells attached significantly more along the wall of the channels. Bacterial attachment remained strong at 8 h compared to control, however, was not as robust as the 6 h time-point.

As we observed that the simulated bacterial cell experienced increased shear stress at higher flow rates (Figures 4C, D), we hypothesized that high flow rates not only reduced bacterial attachment to surfaces but also reduced bacterial biofilm formation in xylem vessels. To test this hypothesis, we seeded CMC-DOPA coated microfluidic devices with GFP-expressing *Rps* GMI1000 and observed the attachment of bacterial cells after 6 h in the devices at different flow rates. We observed that *Rps* cells attached more to the wall of the channels at lower flow rates (0, 10, and 20 μL/h) than higher flow rates (40 and 80 μL/h, Figure 5B). We found that *Rps* biofilm formation was higher at 10 μL/h than 0 μL/h, but was reduced significantly at higher flow rates (20, 40, and 80 μL/h) than at 10 μL/h (Figures 5C, D). Of note, at 80 μL/h flow rate, *Rps* biofilm failed to spread from the entrance of the channels to downstream positions. Surprisingly, *Rps* formed only microcolonies but not complex biofilm at 0 μL/h flow rate. This could be because of the limited amount of nutrient in tomato xylem sap available for *Rps* to grow and form biofilm in the small volume of the channels when there was no flow of fresh xylem sap into the channels over the course of the experiment.

## 4 Discussion

To our knowledge, this study reported for the first time a functional microfluidic system that mimicked the physical and chemical condition of the tomato xylem vessels and facilitates real-time investigations into *Rps* biofilm within a dynamic xylem-like environment. Previous studies on *Rps* biofilm (including our own work) relied on culturing bacterial biofilm in defined media (CPG or minimal medium) on glass and plastic surfaces, or imaging *Rps* in fixed plant tissue, which may limit the biological relevance of the findings (Mori et al., 2016; Tran et al., 2016; Khokhani et al., 2017; Tancos et al., 2018).

First, our system contained 20 parallel channels allowing multiple replicates in one single experiment, similarly to other high-throughput lab-on-chip systems (De La Fuente et al., 2007; Monteiro et al., 2021) (Figures 1A, B). Each channel had a square cross section of 50 × 50 µm (Figure 1B), comparable with the majority of tomato-xylem cross areas (Ingel et al., 2021). The xylem environment was generated by introducing xylem sap collected directly from tomato cultivars to the channels using a syringe pump. While numerous studies employed syringe pumps to regulate flow within microfluidic channels (De La Fuente et al., 2007; Cogan et al., 2013; Monteiro et al., 2021), only few have integrated xylem-like media into their systems (Cogan et al., 2013; Harting et al., 2021) or explored the use of actual xylem sap, owing to its less defined properties (Kandel et al., 2016; Khokhani et al., 2017; Gerlin et al., 2021) and its less favorable conditions for bacterial biofilm formation *in vitro* (Supplementary Figure S2; Figures 3E–H). We, therefore, analyzed the viscosity of various culture media and xylem saps, showing their similarity, which should not alter the flow behavior (Supplementary Figure S4). We validated both experimentally and computationally the laminar flow profile within the channel (Figures 4A–C; Supplementary Figure S5) that emulated the natural flow within the plant xylem (Michelle Holbrook and Zwieniecki, 2011). Our simulation further demonstrated the linear relationship of flow shear stress and drag force applied on a bacterial cell model (Figures 4C–E), which was consistent with other works studying the flow effect on xylem-inhabiting pathogens (De La Fuente et al., 2007; Monteiro et al., 2021). Then, to mimic the chemical component of xylem cell wall, CMC was coated on the channel surfaces through polydopamine surface chemistry (Lee et al., 2007; Lee et al., 2019; Barclay et al., 2017) which provided a robust stable coating material for multiple substrates including glass and PDMS (Figures 3A–D) while retaining both CMC and DOPA unique properties (Figure 2). Dopamine was conjugated with CMC by DMTMM mediated amidation as evidenced by ^1^H-NMR and UV absorbance (Figures 2A–C). The low DS value of DOPA minimized the interference of DOPA modification with CMC bioactivities. Under alkaline conditions, dopamine moiety on CMC-DOPA polymerized to form thin polydopamine films for CMC coating via strong covalent and noncovalent bonds (Hong et al., 2018; Lee et al., 2019; Lee et al., 2020). The channels functionalized with CMC-DOPA coatings showed significant increase in bioactivity. We identified that CMC-DOPA coating at concentration of 20 mg/mL significantly improved the coating efficiency and wettability on both glass and PDMS substrates (Figures 3A–D) and actively promoted *Rps* biofilm formation within the xylem sap environments (Figures 3E–H, 5).

Functionalized microfluidic channels have been proven a powerful tool to dissect the behavior of bacterial pathogens. Modifying microfluidic channels with CMC or the surface adhesin XadA1, for instance, could greatly enhance *Xylella fastidiosa* biofilm formation (Monteiro et al., 2021). Consistent with that study, we herein found that CMC-DOPA coatings significantly enhanced *Rps* biofilm formation (Figures 3E–H). In particular, we observed for the first time that *Rps* could successfully form biofilm in xylem sap condition as compared to no-coating control. These data confirm the importance of compatible substrata for bacterial biofilm formation. Future studies can examine more sophisticated substrata that replicate different xylem cell wall chemical compositions. For example, besides the cellulose and hemicellulose, other polysaccharides such as lignin, pectin were found to increase in maturing vessels (Lowe-Power et al., 2018) and in bacterial resistant cultivars (Ishihara et al., 2012; Kashyap et al., 2022). Therefore, we can synthesize pectin-DOPA and lignin-DOPA via the same amidation reaction (Dai et al., 2019; Diao et al., 2019) and incorporated into CMC-DOPA. Furthermore, polysaccharides from wilt resistant and susceptible tomato cultivars can be used with DOPA chemistry to develop coatings directly from the natural plant cell walls.

The xylem-mimicking system we developed in this study allows for studying multiple *Rps* responses to seeding time and flow rates. For instance, we found that *Rps* cells attached to coated surfaces more with prolonged seeding time (Figure 5A), consistent with the time windows for bacterial attachment on tomato roots (3–6 h) in previous studies (Dalsing and Allen, 2014; Tancos et al., 2018). Interestingly, bacterial attachment at the channel inlets seemed to be optimal at 6 h but not at longer seeding time (8 h, Figure 5A). We speculate that *Rps* attachment could be reversible, and attached cells could detach to find new niches along the channel (Hinsa et al., 2003; Caiazza and O’Toole, 2004; Armbruster and Parsek, 2018). Although replicating the exact structure and topography of the actual xylem remains technically challenging, we were capable of characterizing the flow profiles within the channels and utilized the data for interpolating the bacterial responses (Figure 4; Supplementary Figure S5). Concurring with previous studies, our simulation confirmed that bacterial cells experienced higher drag forces with increasing flow rates (Figures 4C–E) (De La Fuente et al., 2007; Cogan et al., 2013; Monteiro et al., 2021), when we modified the system input flow rate or changed the positions of the bacterial cell model from the mid-wall to the channel corner. At higher flow-rates, *Rps* biofilm was found decreased experimentally, suggesting the impact of increased drag forces on hindering bacterial adhesive forces and the resulting biofilm establishment (Figures 5B–D). Interestingly, we observed that the xylem sap flow rate of resistant cultivar Hawaii 7996 was significantly higher than that of the susceptible cultivar Bonny Best (Supplementary Figure S3). It is possible that in resistant Hawaii 7996 xylem vessels, *Rps* cells would have to overcome a higher drag force to establish its initial attachment, thus limiting bacterial colonization (Caldwell et al., 2017) and reducing the resulting biofilm that could block the vessels causing wilt symptoms. In addition to examining the effect of xylem sap flow rate on *Rps* behavior, the system can be used to explore various flow effects on single-cell and multicellular responses. These include changes in cell shapes and motility during colonization, bacterial proliferation events, early stages of biofilm formation, matured biofilm architecture and the resulting hydraulic resistance (Dreszer et al., 2013; Persat et al., 2015; Straub et al., 2020).

Many studies attempted to elucidate the molecular mechanisms that govern *Rps* attachment, motility, and biofilm formation (Lowe-Power et al., 2018). *Rps* upregulated many adhesion proteins (adhesins), including adhesins RcpA and RcpB at early stages of infection on the root surfaces and in the endosphere (Khokhani et al., 2017; Carter et al., 2023). The adhesin RadA, together with RcpA and RcpB, allowed for efficient bacterial attachment to abiotic and root surfaces, as well as contributed to bacterial fitness in tomato stems (Carter et al., 2023). Inside the tomato stems, *Rps* lost its flagellar motility (Meng et al., 2011; Kai, 2023) to form complex biofilm composed of EPS and extracellular DNA (Tran et al., 2016), although a subpopulation of *Rps* might retain motility as a means to disperse from mature biofilm. It was also speculated that the spread of *Rs* in xylem vessels was mainly due to its twitching motility mediated by type IV pili (Kang et al., 2002; Wairuri et al., 2012; Siri et al., 2014). The knowledge obtained on these factors that contribute to biofilm formation, however, is mostly from disease assays and *in vitro* biofilm experiments on conventional abiotic surfaces. The xylem-mimicking system that we developed, therefore, presented a unique opportunity to advance knowledge on *Rps* pathogenesis via real-time imaging and quantification. One limitation of our system is the time- and space-consuming process of collecting sufficient tomato xylem sap, which could be overcome by the development of defined media that mimics the sap biochemistry (Khokhani et al., 2017; Gerlin et al., 2021). The system can be further optimized and integrated with molecular and imaging techniques, such as Tn-Seq (Georgoulis et al., 2021), promoter probing (Miller et al., 2000), and live-cell imaging (De La Fuente et al., 2007; Kozgunova and Goshima, 2019) to unravel novel molecular mechanisms involved in biofilm formation and vessel blockage of other xylem-inhabiting pathogens, such as *Xylella fastidiosa*, *Clavibacter michiganensis*, *Erwinia amylovora*, and *Erwinia tracheiphila*, etc. It is estimated that 40%–80% prokaryotes on Earth exist in the form of biofilm (Flemming and Wuertz, 2019). Understanding the biology of biofilm, especially in plant pathogenic microbes, could result in effective controls of these pathogens.

## 5 Conclusion

In conclusion, this study shows a novel *in vitro* system developed based on microfluidics technology and polydopamine surface mimicry that could provide more biologically relevant outputs. We have demonstrated the application of the system for studying how *R. pseudosolanacearum* cells adhere and form biofilm in flow conditions. The system not only offers a potential means for real-time visualization and quantification of various plant pathogenic biofilms in xylem-mimicking condition, it can also allow multiple examinations of different flow-related variables affecting the bacterial adhesion, motility, growth and biofilm development. As we established our *in vitro* system for biofilm examination, we can further investigate the effect of various fitness factors that directly contribute to bacterial colonization and biofilm formation under conditions that mimic xylem flow and the plant cell-wall. These factors include, but are not limited to, adhesion factors such as flagella, pili, nucleases, adhesins, and cell-wall modifying enzymes such as expansins, cellulases, polygalacturonases. Gaining more insights from these findings will enable us to elucidate the mechanisms by which many xylem-inhabiting bacteria adapt to the dynamic xylem environment and to suggest prevention approaches against bacterial wilt diseases.

## Data Availability

The raw data supporting the conclusion of this article will be made available by the authors, without undue reservation.
