# Supplementary material for: Development of a tomato xylem-mimicking microfluidic system to study Ralstonia pseudosolanacearum biofilm formation
Source: Front Bioeng Biotechnol. 2024 May 27;12:1395959. doi: 10.3389/fbioe.2024.1395959 (PMC11163092; doi:10.3389/fbioe.2024.1395959)
Supplement: Supplementary file 1 [file DataSheet1.pdf]

Supplementary Materials

1 Supplementary Methods

Method S1: Biofilm assay on PVC plate

Overnight culture of *Rps* GMI1000 was grown in CPG broth at 28°C. Bacterial cells were collected by centrifugation, resuspended in the same volume of sterile water and adjusted to OD_{600nm}=0.1 in either CPG broth or Bonny Best xylem sap. 200 µl of this bacterial suspension was seeded into wells of PVC microplates (N=10). The plates were incubated at 28°C for 24 hr, then stained with 1% Crystal Violet for 20 min before washing three times with sterile water. Crystal Violet embedded in *Rps* biofilm was then resuspended in 95% Ethanol, transferred to a new Polystyrene plate. OD_{570nm} was measured in a plate reader (Biotek Synergy H1) and normalized to bacterial growth (OD_{600nm}) measured from a duplicated plate started at the same time as the original PVC biofilm plate. The experiments were repeated twice with similar results.

Method S2: Measurement of xylem sap flow rates

Xylem sap flow rates were measured from volumes of sap recovered from decapitated tomato plants. Briefly, Bonny Best and Hawaii 7996 tomato seeds were sown on MiracleGro potting mix and grown for two weeks at 30°C. Five-week-old tomato plants were decapitated right above the cotyledons. Xylem sap in the first 2-3 min were discarded and the stumps were wiped with Kimwipes to remove cell debris and phloem sap contamination. Xylem sap was collected for a period of three hours, then the volume was measured to determine the amount of sap produced by each cultivar. Xylem sap was then filter-sterilized with a 0.22-µm filter, aliquoted into 1.5-ml microcentrifuge tubes and kept frozen at -80°C until needed. The experiment was repeated twice on two different batches of Bonny Best and Hawaii 7996 tomato plants (N≥20 plants) with similar results.

Method S3: Viscosity evaluation of different xylem saps and culture media

Measurement of the fluid viscosity was performed using a rheometer (Anton Paar, MCR 702 eSpace). Different fluid samples included cell culture media (Basic minimal medium - BMM, and Cassamino acid-Peptone-Glycerol medium - CPG), xylem saps from two tomato cultivars (susceptible cultivar - Bonny Best, and resistant cultivar - Hawaii 7996), and deionized water. The rheometer was configured with a parallel plate (PP25 with 25 mm in diameter) at a horizontal position and initial gap of 20 mm to define a sample volume of 0.7 ml. The temperature was adjusted from 20ºC to 40ºC with a 5ºC interval. To start, a fluid sample without air bubbles was loaded onto the measuring plate center. The top rotating plate was lowered onto the sample with a zero-gap set at 1mm. Excessive volume of sample was trimmed with Kimwipes® wiper. Rotational shear rate was then applied from 0 to 300 s^{-1}. Measurement data of 50 points were collected at each temperature. Deionized water was used as a control. Raw data was recorded on RheoCompass™ software then exported for analysis. The datasets for each sample were reported as viscosity-shear profiles. Average viscosities and SEMs for all samples at different temperatures were calculated.

2 Supplementary Data

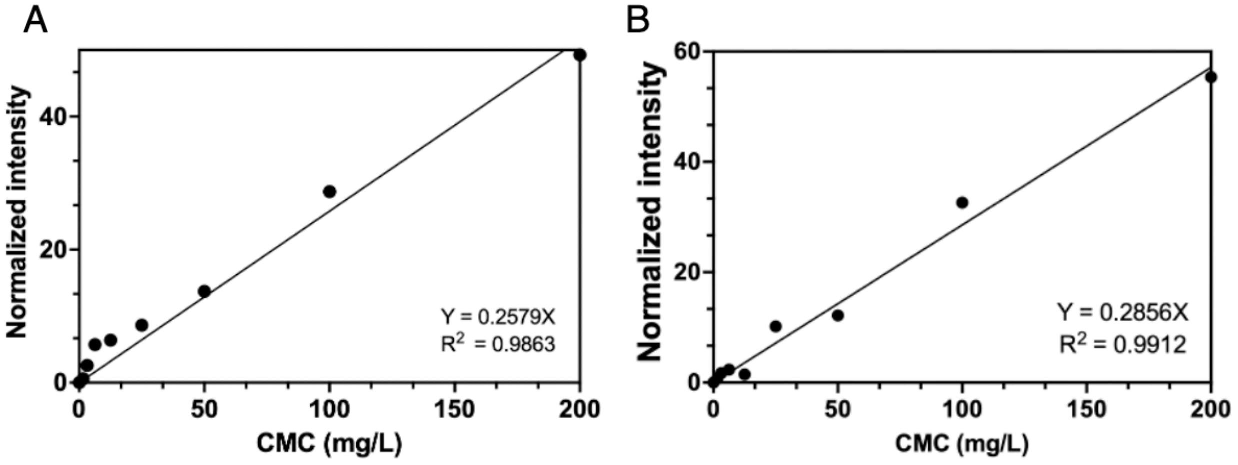

Figure S1: Standard curve of different CMC concentrations generated from CFW assay and fluorescent intensity measurement. The average of mean intensity at each concentration was normalized to one at 0 mg/L (i.e. DI water) concentration. (A) Glass surface (B) PDMS surface

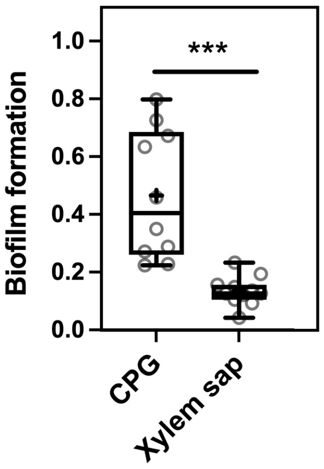

Figure S2: **Biofilm assay on PVC plate.** Biofilm formation was measured as OD_{570nm}/OD_{600nm} from Crystal Violet biofilm assay. *Rps* GMI1000 was allowed to form biofilm in either rich medium (CPG) or Bonny Best xylem sap in 24 hr at 28°C. The absorbance of Crystal Violet (OD_{570nm}) was measured with a microtiter plate reader and normalized to bacterial growth (OD_{600nm}).

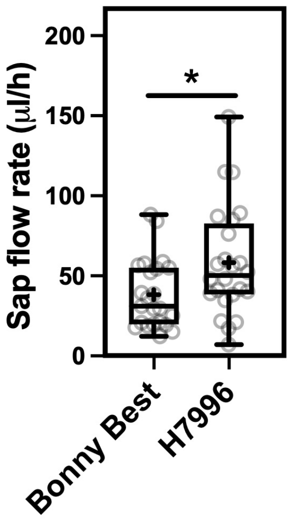

Figure S3: **Xylem sap flow rates** of susceptible (Bonny Best) and resistant (Hawaii 7996) were collected from five-week old tomato plants. Sap flow rate was determined by the amount of sap collected over a period of three hours from cut stems. The cut stem was wiped once with Kimwipes before collecting sap to remove cellular debris and contamination of phloem liquid.

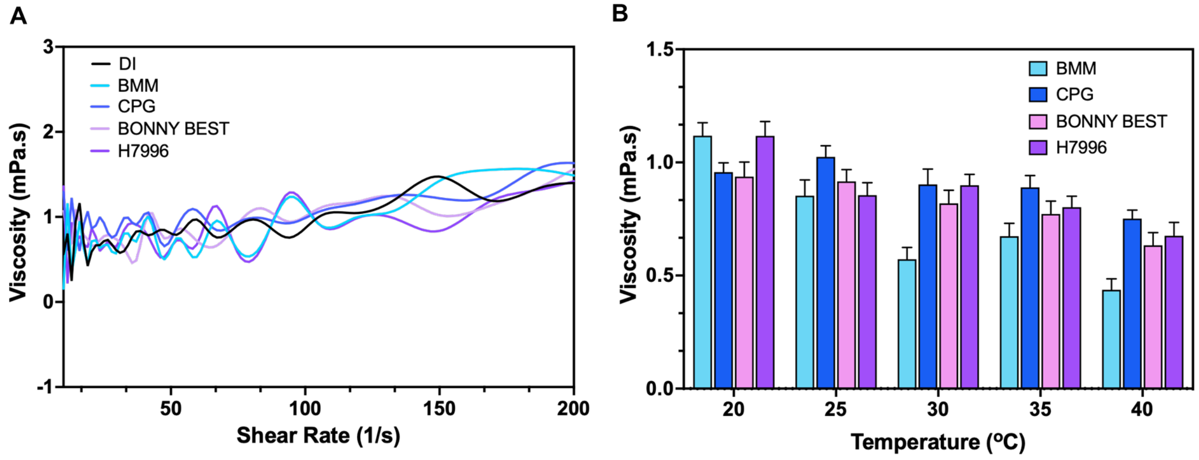

Figure S4: **Viscosities of different fluids** used in the microfluidic system. (A) Viscosity-shear profiles at 25°C of culture media (BMM and CPG), xylem saps (Bonny Best and Hawaii 7996) and deionized water (DI) showing Newtonian fluids, of which viscosity is constant and independent of shear rates. (B) Changes in viscosities of the culture media and xylem saps at varied temperatures.

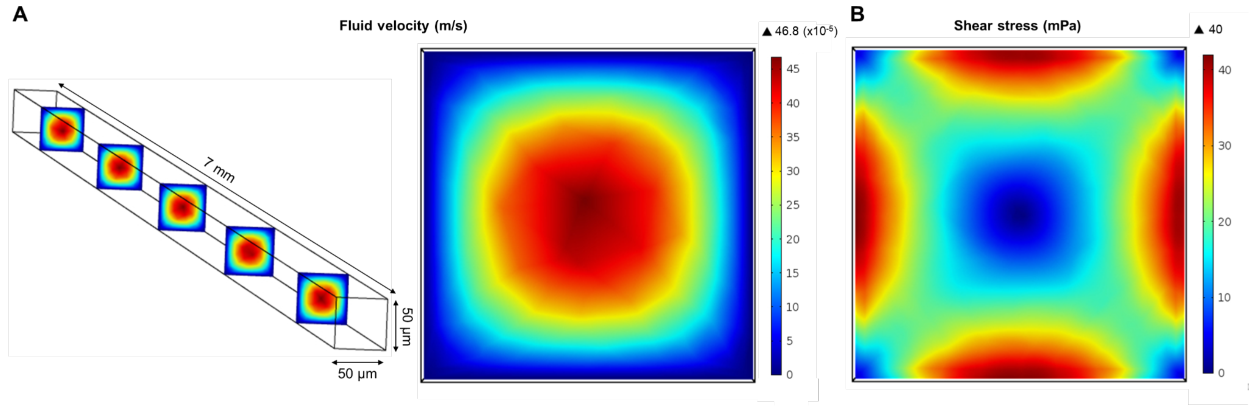

Figure S5: Flow profile simulation in the channel using COMSOL multiphysics software. (A) Velocity profile throughout the whole channel and of a cross section. (B) Corresponding shear stress of a channel cross section. Inlet flow rate preset at 2 µl/hr (equivalent to system flow rate of 40 µl/hr).
